# Ventilatory Responses to Progressive Treadmill Speeds in Women: A Comparative Analysis of Nasal, Oral, and Oronasal Breathing Conditions

**DOI:** 10.3390/ijerph22050718

**Published:** 2025-05-01

**Authors:** Seung Hee Lee, Yongsuk Seo, Dae Taek Lee

**Affiliations:** Exercise Physiology Laboratory; Kookmin University, Seoul 02707, Republic of Korea; seung@kookmin.ac.kr (S.H.L.); yseokss@kookmin.ac.kr (Y.S.)

**Keywords:** ventilatory responses, exercise, respiration, female

## Abstract

Background: Breathing conditions influence ventilatory efficiency and exercise performance, but little research has examined how different breathing conditions affect cardiorespiratory responses in women. Despite the growing popularity of nasal-only breathing in fitness culture, its physiological benefits remain unclear. The purpose of the current study is to examine the ventilatory responses to nasal, oral, and oronasal breathing during treadmill exercise at speeds of 5 to 11 km/h in 10 healthy females. Methods: Participants completed sessions under each breathing condition while heart rate (HR), oxygen uptake (VO_2_), ventilatory equivalent for CO_2_ (VE/VCO_2_), respiratory frequency (Rf), tidal volume (VT), minute ventilation (VE), and respiratory timing variables were measured. Results: Breathing condition had minimal impact at lower speeds (5–7 km/h). However, at higher intensities (10–11 km/h), nasal breathing resulted in lower Rf and VE but elevated VE/VCO_2_, indicating reduced ventilatory efficiency. In contrast, oral and oronasal breathing facilitated greater VE and shorter inspiratory and expiratory times, supporting ventilation under vigorous exercise. Conclusions: While nasal breathing may suffice at low intensities, it is inadequate at higher intensities, potentially leading to carbon dioxide accumulation and early fatigue. These findings support the use of oral or oronasal breathing during higher-intensity activity and highlight the need for individualized breathing strategies.

## 1. Introduction

With urbanization projected to increase by 2.5 billion people between 2018 and 2050 [1], more individuals are engaging in physical activity outdoors, including walking, running, and cycling. As these activities become more popular, attention has shifted toward breathing behaviors during exercise. This is not only because of their influence on performance and comfort, but also due to increasing concerns about exposure to environmental pollutants such as particulate matter (e.g., PM2.5 and PM10) during outdoor activity [2]. In recent years, various fitness trends have promoted specific breathing conditions such as exclusive nasal breathing based on claims of improved endurance, reduced stress, and enhanced oxygen utilization [3,4]. However, many of these claims remain anecdotal or are based on limited empirical evidence. Studies have shown that overly restrictive breathing conditions may compromise ventilation and gas exchange, particularly at higher intensities, leading to adverse outcomes such as carbon dioxide (CO_2_) retention, dizziness, and reduced performance [5,6]. These risks may be especially pronounced in unsupervised or self-directed exercise contexts where individuals adopt such breathing without professional guidance.

Breathing occurs through nasal, oral, or a combination of both pathways (oronasal), with each mode serving distinct physiological functions depending on activity and environmental demands [7,8]. At rest, nasal breathing is predominant, allowing for filtration, warming, and humidification of inspired air [9,10]. As exercise intensity increases, however, most individuals naturally shift toward oral or oronasal breathing to reduce airway resistance and support greater ventilatory demand [4,11]. This transition often coincides with the ventilatory threshold, the point at which CO_2_ clearance becomes inefficient through nasal breathing alone [12,13].

Previous studies have explored the effects of different breathing conditions across a range of populations and exercise contexts. For example, Recinto et al. (2017) found that oral breathing enhanced anaerobic power output compared to nasal breathing in healthy young adults [4], while LaComb et al. (2017) reported that nasal breathing may be less effective at higher intensities during submaximal aerobic exercise [3]. In elite endurance athletes, Lucía et al. (1999) observed a natural shift from nasal to oronasal breathing as exercise intensity increased [14]. However, most of this research has been conducted in male or mixed-gender cohorts, and relatively few studies have evaluated how different breathing strategies affect ventilatory efficiency in healthy women. To address this gap, the present study investigates the cardiorespiratory responses to nasal, oral, and oronasal breathing in a female population. Although these physiological adaptations are well documented in male-dominated samples, research on women remains limited. This underrepresentation is noteworthy, as women typically have smaller airways, lower lung volumes, and distinct ventilatory control mechanisms compared to men [5,15,16,17,18,19]. Understanding how breathing condition influences exercise responses in women may help optimize training approaches and improve safety.

Although numerous studies have assessed cardiorespiratory responses to exercise, most have focused on a single breathing mode, typically oral or oronasal breathing, without systematically comparing multiple breathing routes under controlled conditions to isolate their specific influence [3,4,14]. Only a limited number of studies have directly contrasted nasal, oral, and oronasal breathing during exercise, and these have predominantly involved male or mixed-gender samples. To date, there is a lack of research comprehensively evaluating ventilatory responses across all three breathing conditions within the same individuals, particularly in women. This highlights the need for controlled, within-subject studies to clarify how breathing condition influences gas exchange and ventilatory efficiency during exercise. Therefore, this study examined how nasal, oral, and oronasal breathing affect ventilatory responses during treadmill exercise at progressively increasing speeds. It was hypothesized that nasal breathing would result in distinct ventilatory adjustments compared to oral and oronasal breathing, particularly at higher exercise intensities, potentially limiting in minute ventilation/carbon dioxide production during nasal breathing.

## 2. Materials and Methods

The current study employed a repeated measure, within-subjects design with counterbalanced order across participants using a Latin square design. This ensured that each breathing condition (nasal, oral, or oronasal) occurred equally. Participants reported to the laboratory on four occasions (familiarization and three experimental trials), separated by at least 7 days to ensure full recovery. During the familiarization session, participants underwent prescreening, anthropometric measurements, and were instructed on the protocol and instrumentation. Experimental trials involved breathing through one of three conditions: (1) nasal, (2) oral, or (3) oronasal breathing.

During each trial, participants were instructed to breathe exclusively through one assigned condition. For nasal breathing, participants were thoroughly instructed and supervised throughout the trial to ensure compliance. In oral breathing trials, participants were instructed to keep their mouths open and avoid nasal inhalation with a nose clip. For oronasal conditions, no restriction was imposed. Researchers closely monitored breathing, and trials were terminated if compliance could not be maintained. Each participant completed a treadmill protocol consisting of 2-min stages at increasing speeds: 5, 7, 9, 10, and 11 km/h. These speeds were selected to reflect a progression from light to vigorous intensity, allowing observation of ventilatory changes as exercise demand increased.

### 2.1. Participants

Ten healthy female participants volunteered to participate in the current study (Table 1). All participants were physically active recreational exercisers who reported engaging in aerobic or resistance-based activities at least 2–3 times per week but were not competitive athletes. Physical activity status was self-reported during pre-screening and confirmed during the familiarization session. Prior to participation, they were screened with medical questionnaires and familiarized with the study protocol. Participants were excluded if they had a history of smoking, current cardiovascular or musculoskeletal disease, or were taking any medication that could affect cardiorespiratory function.

All participants completed a standardized medical history questionnaire and were screened using the Physical Activity Readiness Questionnaire (PAR-Q+) to identify any underlying health conditions or risk factors that could contraindicate moderate-to-vigorous physical activity. Individuals who did not meet the health criteria were excluded from the study. Prior to participation, all eligible participants received both written and verbal explanations of the study procedures and associated risks and provided written informed consent in accordance with ethical guidelines.

All participants received written and verbal explanations of the study procedures and risks, and provided written informed consent in accordance with ethical guidelines. The study was approved by the Institutional Review Board of Kookmin University (IRB No. KMU-202210-HR-338; approval date: 13 January 2023). The sample size was determined via G*power software (ver. 3.1.9.2) with an assumed power of 0.8 and an effect size (Cohen’s d = 0.7) based on preliminary data from a pilot study involving three participants. While this effect size represents a moderate-to-large difference and allowed for a feasible recruitment target (*n* = 10), we acknowledge that detecting smaller effects (e.g., d = 0.25) would require a substantially larger sample. It was concluded that recruiting ten participants would be necessary to attain statistical significance of the ventilatory response among the breathing conditions.

### 2.2. Procedures

Before participating, participants were familiarized with the study protocol. A detailed explanation of the purpose and procedures of the study was provided. Subsequently, upon understanding the study, participants provided both written and verbal informed consent to participate.

Each participant visited the laboratory on four separate occasions, with at least 7 days between visits to ensure recovery and eliminate residual fatigue effects. The first visit was a familiarization session, while the remaining three visits served as experimental trials under each assigned breathing condition. All experimental sessions were conducted at the Exercise Physiology Laboratory of Kookmin University at the same time of day to minimize the influence of circadian variation. Only one participant was evaluated per time slot per day to ensure individualized attention and consistent experimental control. The laboratory was maintained at a controlled temperature (21–23 °C) and relative humidity (40–50%). Participants were instructed to avoid caffeine, alcohol, and vigorous exercise for at least 24 h prior to testing.

During the familiarization session, participants were introduced to the treadmill (T170 DE, COSMED, Fridolfing, Germany) and portable metabolic cart (COSMED K5, Rome, Italy), and practiced using each of the three breathing conditions. Resting measurements of height (DS-102, JENIX, Seoul, Republic of Korea) and body mass (DB-150, CAS, Yangju-si, Gyeonggi-do, Republic of Korea) were also recorded. The Physical Activity Readiness Questionnaire (PAR-Q+) and a medical history questionnaire were administered to assess eligibility.

On the day of the experimental trials, participants were dressed in a t-shirt, shorts, and self-selected athletic shoes. After approximately 5 min of rest, baseline measurement of heart rate (HR) was monitored with an HR monitor (Polar RS8000 CS, Polar Electro Oy, Kempele, Finland), and oxygen uptake (VO_2_) was assessed using a portable metabolic system. Following baseline measurements, participants walked or ran on the treadmill (T170 DE, Cosmed, Fridolfing, Germany) at an ordered speed of 5, 7, 9, 10, and 11 km/h for 2 min at each speed, with one of the three breathing conditions.

These speeds were selected to represent a range from low to vigorous intensity while avoiding exhaustion. A maximum speed of 11 km/h was used rather than 12 km/h because pilot testing revealed that nasal breathing could not be sustained at or beyond 12 km/h for the majority of participants without excessive compensatory behavior or protocol termination. This upper speed was selected to ensure data consistency and participant safety while still inducing physiological stress sufficient to challenge ventilatory function.

Participants were instructed to use only the designated breathing condition throughout each trial. For nasal breathing, participants kept their mouths closed under supervision. For oral breathing, nasal inhalation was discouraged. For oronasal breathing, no restrictions were imposed. Breathing compliance was monitored in real-time by trained researchers, and any deviation resulted in trial termination or repetition.

During the exercise, HR, VO_2_, minute ventilation/carbon dioxide production (VE/VCO_2_), respiratory frequency (Rf), tidal volume (VT), minute ventilation (VE), inspiratory time (Ti), expiratory time (Te), and total respiratory cycle time (Ttot) were measured. VCO_2_ was not directly measured; VE/VCO_2_ values were obtained via the metabolic system’s internal calculations.

### 2.3. Data Analysis

Using the Statistical Package for the Social Sciences 22.0 (IBM-SPSS, Somers, NY, USA), a two-way (breathing conditions by speed) repeated measure analysis of variance (ANOVA) was utilized for HR, VO_2_, VCO_2_, VE/VCO_2_, Rf, VT, VE, Ti, Te, and Ttot. Prior to analysis, the normality of each variable was assessed using the Shapiro–Wilk test. When a significant F ratio for a main effect or interaction was detected, post hoc pairwise comparisons were conducted using the Bonferroni correction to limit Type I errors from multiple testing. Partial eta squared (η^2^) was reported as a measure of effect size for all main effects and interactions, with values interpreted as small (0.01), medium (0.06), and large (≥0.14) based on conventional thresholds. Statistical significance was set at *p* ≤ 0.05 and all data are presented as mean ± standard deviation (SD).

## 3. Results

### 3.1. Cardiopulmonary Responses

**Figure 1:**
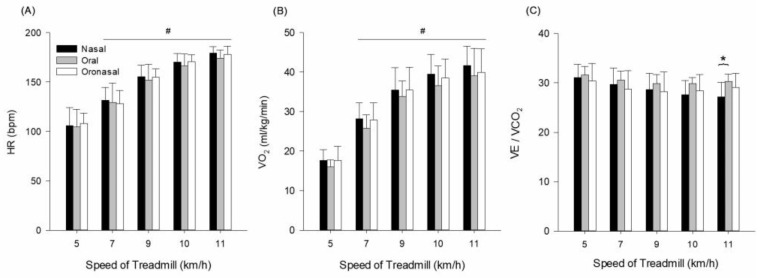
(**A**) HR—heart rate, (**B**) VO_2_—oxygen uptake, and (**C**) VE/VCO_2_—minute ventilation/carbon dioxide production response during exercise with three different breathing conditions. # Significantly different from 5 km/h. * Significantly different from nasal breathing.

Measurements of HR, VO_2_, and VE/VCO_2_ are presented in Figure 1 and Table 2. HR indicated a main effect for condition (F = 231.613, *p* ≤ 0.001, $\eta_{p}^{2}\;$ = 0.971), speed (F = 27.501, *p* ≤ 0.001, $\eta_{p}^{2}\;$ = 0.797), and interaction (F = 6.026, *p* ≤ 0.001, $\eta_{p}^{2}\;$ = 0.463). Pairwise comparison indicates that HR gradually increases over speed compared to 5 km/h (*p* ≤ 0.05) (Figure 1A).

VO_2_ indicated a main effect for condition (F = 223.635, *p* ≤ 0.001, $\eta_{p}^{2}$ = 0.970), main effect for speed (F = 41.666, *p* ≤ 0.001, $\eta_{p}^{2}$ = 0.856), and interaction (F = 11.221, *p* ≤ 0.001, $\eta_{p}^{2}\;$ = 0.616). The significant interaction suggests that VO_2_ increased more steadily with oral and oronasal breathing across speeds, while nasal breathing showed a plateau effect at higher intensities. Pairwise comparison indicates that VO_2_ gradually increases over speed compared to 5 km/h (*p* ≤ 0.05) (Figure 1B).

VE/VCO_2_ indicated a main effect for condition (F = 8.505, *p* = 0.004, $\eta_{p}^{2}$ = 0.549), no main effect for speed (F = 2.046, *p* = 0.115, $\eta_{p}^{2}\;$ = 0.226), and interaction (F = 1.219, *p* = 0.305, $\eta_{p}^{2}\;$ = 0.148).

This result indicates that VE/VCO_2_ differed by breathing condition regardless of exercise intensity. However, a notable difference was observed at 11 km/h, where nasal breathing showed higher VE/VCO_2_ compared to oral breathing (*p* = 0.011) (Figure 1C). This may suggest that nasal breathing was less effective at removing CO_2_ at higher intensities, potentially due to limited ventilatory capacity.

### 3.2. Pulmonary Ventilatory Responses

**Figure 2:**
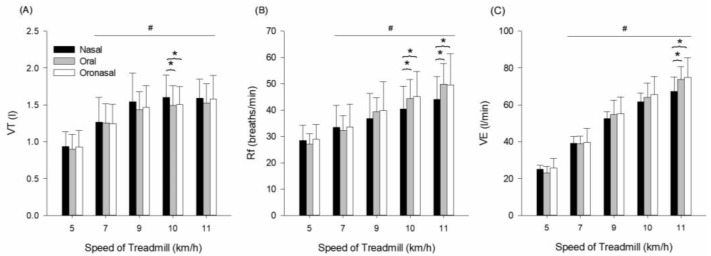
(**A**) VT—tidal volume, (**B**) Rf—respiratory frequency, and (**C**) VE—minute ventilation response during exercise with three different breathing conditions. # Significantly different from 5 km/h. * Significantly different from nasal breathing.

Measurements of VT, Rf, and VE are presented in Figure 2 and Table 3. VT indicated a main effect for condition (F = 69.598, *p* ≤ 0.001, $\eta_{p}^{2}\;$ = 0.909), speed (F = 18.124, *p* ≤ 0.001, $\eta_{p}^{2}\;$ = 0.721), and interaction (F = 6.108, *p* ≤ 0.001, $\eta_{p}^{2}\;$ = 0.466). This interaction indicates that the increase in tidal volume across treadmill speeds was more pronounced for nasal breathing up to 10 km/h, after which it leveled off, while oral and oronasal breathing conditions exhibited less consistent changes. Pairwise comparison indicates that VT gradually increases over speed compared to 5 km/h (*p* ≤ 0.05). At 10 km/h, VT was significantly lower during oronasal breathing (*p* = 0.006) and oral breathing (*p* = 0.008) compared to nasal breathing (Figure 2A).

Rf indicated a main effect for condition (F = 60.782, *p* ≤ 0.001, $\eta_{p}^{2}\;$ = 0.897), speed (F = 21.850, *p* ≤ 0.001, $\eta_{p}^{2}\;$ = 0.757), and no interaction (F = 0.865, *p* = 0.551, $\eta_{p}^{2}\;$ = 0.110). Pairwise comparison indicates that Rf gradually increases over speed compared to 5 km/h (*p* ≤ 0.05) (Figure 2B). At 10 km/h, Rf was significantly higher during oronasal breathing (*p* = 0.006) and oral breathing (*p* = 0.045) compared to nose breathing. At 11 km/h, Rf was significantly higher during oronasal breathing (*p* = 0.008) and oral breathing (*p* = 0.014) compared to nose breathing. VE indicated a main effect for condition (F = 336.886, *p* ≤ 0.001, $\eta_{p}^{2}\;$ = 0.980), speed (F = 75.811, *p* ≤ 0.001, $\eta_{p}^{2}\;$ = 0.915), and interaction (F = 5.224, *p* ≤ 0.001, $\eta_{p}^{2}\;$ = 0.427). The interaction indicates that nasal breathing could not sustain proportional increases in ventilation at higher speeds, whereas oral and oronasal breathing continued to support the ventilatory demand. Pairwise comparison indicates that VE gradually increased over speed compared to 5 km/h (*p* ≤ 0.05). At 11 km/h, VE was significantly higher during oronasal breathing (*p* = 0.029) and oral breathing (*p* = 0.005) compared to nasal breathing (Figure 2C).

### 3.3. Breathing Timing Responses

**Figure 3:**
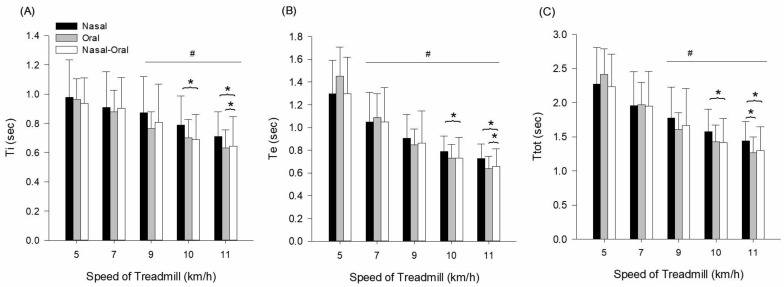
(**A**) Ti—inspiratory time, (**B**) Te—expiratory time, and (**C**) Ttot—total respiratory cycle time response during exercise with three different breathing conditions. # Significantly different from 5 km/h. * Significantly different from nasal breathing.

Measurements of Ti, Te, and Ttot are presented in Figure 3 and Table 4. Ti indicated a main effect for condition (F = 27.030, *p* ≤ 0.001, $\eta_{p}^{2}\;$ = 0.794), main effect for speed (F = 7.297, *p* ≤ 0.001, $\eta_{p}^{2}\;$ = 0.510), and no interaction (F = 0.640, *p* = 0.740, $\eta_{p}^{2}\;$ = 0.084). Ti gradually decreased over speed compared to 5 km/h (*p* ≤ 0.05). At 10 km/h, Ti was significantly lower during oronasal breathing (*p* = 0.004) compared to nose breathing. At 11 km/h, Ti was significantly lower during oronasal breathing (*p* = 0.007) and oral breathing (*p* = 0.044) compared to nose breathing (Figure 3A).

Te indicated a main effect for condition (F = 83.978, *p* ≤ 0.001, $\eta_{p}^{2}\;$ = 0.923), main effect for speed (F = 24.701, *p* ≤ 0.001, $\eta_{p}^{2}\;$ = 0.779), and interaction (F = 4.354, *p* = 0.001, $\eta_{p}^{2}\;$ = 0.383). The interaction indicates that Te decreased more steeply with oral and oronasal breathing compared to nasal breathing at higher intensities. Te gradually decreased over speed compared to 5 km/h (*p* ≤ 0.05). At 10 km/h, Te was significantly lower during oronasal breathing (*p* = 0.043) compared to nasal breathing. At 11 km/h, Te was significantly lower during oronasal breathing (*p* = 0.010) and oral breathing (*p* = 0.007) compared to nasal breathing (Figure 3B).

Ttot indicated a main effect for condition (F = 59.582, *p* ≤ 0.001, $\eta_{p}^{2}\;$ = 0.895), main effect for speed (F = 19.838, *p* ≤ 0.001, $\eta_{p}^{2}\;$ = 0.739), and no interaction (F = 1.950, *p* = 0.070, $\eta_{p}^{2}\;$ = 0.218). Ttot gradually decreased over speed compared to 7 km/h (*p* ≤ 0.05). At 10 km/h, Ttot was significantly lower during oronasal breathing (*p* = 0.008) compared to nasal breathing. At 11 km/h, Ttot was significantly lower during oronasal breathing (*p* = 0.004) and oral breathing (*p* = 0.009) compared to nasal breathing (Figure 3C).

## 4. Discussion

This study evaluated the impact of different breathing conditions on ventilatory responses during progressive treadmill exercise in women. As exercise intensity increased, nasal breathing became less effective in maintaining adequate ventilation, particularly at 10 to 11 km/h. This likely prompted a compensatory shift to oral or oronasal breathing, enabling greater CO_2_ clearance and ventilatory support.

Although VO_2_ and HR increased with speed as expected [20,21], no significant differences were observed in VO_2_ across breathing conditions, suggesting that oxygen delivery was maintained regardless of the condition [22,23]. However, HR remained similar across conditions, contrasting with studies reporting reduced sympathetic activity and lower HR during nasal breathing [24,25]. This discrepancy may be attributed to differences in nasal breathing protocol duration and participants’ habituation levels, both known to influence autonomic responses [3,26,27].

Ventilatory indicators such as Rf and VE increased progressively with exercise intensity, particularly during oral and oronasal breathing. At 11 km/h, VE was significantly higher in oral and oronasal conditions, while nasal breathing showed a relative increase in VE/VCO_2_, indicating reduced ventilatory efficiency under higher physiological demands. This supports previous findings that nasal breathing becomes limited during high-intensity exercise due to airflow resistance and anatomical constraints [3,5,25]. Although direct measurements of CO_2_ accumulation were not obtained, an elevated VE/VCO_2_ ratio is generally interpreted as a marker of ventilatory inefficiency, where ventilation becomes disproportionately high relative to CO_2_ elimination [28,29]. In such cases, a mismatch between CO_2_ production by working muscles and its clearance through respiration may occur, potentially contributing to sensations of dyspnea or early fatigue [30]. In this context, accumulation refers to a mismatch between the rate at which carbon dioxide is produced by working muscles and the rate at which it is expelled through respiration. When ventilation is insufficient, CO_2_ can build up in the bloodstream, potentially triggering symptoms such as breathlessness, dizziness, or the early onset of fatigue [30]. Although certain breathing strategies show promise for ventilatory efficiency and metabolic regulation in specific scenarios (e.g., low-intensity training, clinical populations), scientific validation remains mixed and context-dependent [12,31]. The higher VE/VCO_2_ observed during nasal breathing at elevated intensities may reflect a compensatory response to overcome ventilatory constraints, rather than direct evidence of CO_2_ retention [11,26].

As exercise intensity increased, both inspiratory time (Ti) and expiratory time (Te) demonstrated a gradual reduction across all breathing conditions. The most notable transition occurred at 10 km/h and above, where Ti, Te, and total respiratory cycle time (Ttot) were significantly shortened during oral and oronasal breathing compared to nasal breathing. This suggests a shift toward rapid, shallow breathing patterns to accommodate increased ventilatory requirements, consistent with previously described ventilatory thresholds [27,31]. These results align with the concept of a ventilatory switching point, often observed when ventilation exceeds 35–40 L/min, prompting a shift from nasal to oral breathing [27]. Our study reinforces this threshold, particularly in recreationally active females, who may have lower nasal flow capacity compared to trained individuals [5,31]. Furthermore, it is likely that participants were transitioning across different metabolic intensity domains as speed increased, shifting from predominantly oxidative metabolism to greater glycolytic contribution [31,32]. Since no individualized pre-assessment (e.g., ventilatory or lactate threshold testing) was conducted, participants may have experienced variable physiological demands at comparable treadmill speeds. This metabolic variability could have influenced the timing and extent of breathing condition adjustments observed in this study. Previous studies have suggested that, in healthy adults, metabolic energy costs are typically optimized at walking speeds around 1.4 m/s (5 km/h); however, this value can vary depending on individual characteristics such as sex, fitness level, and body composition, and may not precisely reflect the optimal pace for our study population [32].

Importantly, participants were unable to sustain nasal breathing beyond 11 km/h, indicating the limitations of nasal-only breathing at higher intensities. This finding carries practical implications, especially given the recent promotion of nasal breathing within fitness culture as a superior method for oxygen utilization and endurance enhancement [12]. While nasal breathing may offer benefits at lower intensities or in polluted environments, strict adherence at higher intensities could impair performance by limiting ventilation and CO_2_ clearance, potentially increasing risks such as dizziness, early fatigue, or fainting [30]. These results emphasize the need for caution when adopting fitness trends that impact physiological functions without sufficient scientific validation.

A notable strength of this study is demonstrated by the integration of both physiological understanding and practical implication. By focusing specifically on women, who are often underrepresented in high-performance exercise physiology research, this study provides valuable sex-specific data that can inform safer and more effective training recommendations. As urban populations grow and environmental pollution intensifies, optimizing breathing strategies tailored to both context and individual characteristics may enhance exercise safety, adherence, and overall public health. Furthermore, the fixed-intensity design enabled clear comparisons of ventilatory responses, particularly relevant for individuals engaging in outdoor physical activity under environmental stressors. The observation that nasal breathing at intensities around 10 km/h may contribute to CO_2_ accumulation, as indicated by elevated VE/VCO_2_, underscores the importance of individualized breathing strategies to mitigate increased respiratory effort and potential health risks [2]. Finally, future research should explore how nasal training, anatomical differences, and environmental factors such as pollution, heat, or altitude influence breathing transitions, and aim to establish evidence-based guidelines that account for both benefits and limitations of nasal breathing across various exercise contexts.

### Limitation

These findings suggest that a shift toward oral or oronasal breathing begins to emerge at exercise intensities around 10 km/h. However, since this study evaluated speeds only up to 11 km/h, conclusions about breathing patterns beyond this range remain speculative. Future studies are encouraged to investigate ventilatory responses at higher exercise intensities, including speeds exceeding 12 km/h, to better understand how breathing strategies continue to adapt under increased physiological demand.

This study has several limitations. First, all trials were conducted on a treadmill in a controlled laboratory environment, which may not fully replicate the biomechanical and psychological characteristics of overground exercise [32,33]. Second, the short exposure duration (2 min per speed) limited the assessment of fatigue-related or long-term ventilatory adaptations. Third, the small, homogenous sample of 10 young, recreationally active females restricts generalizability to other populations. Additionally, the absence of respiratory muscle activity or subjective effort measurements (for example, using EMG or Borg ratings) limits interpretation of whether changes in inspiratory and expiratory durations reflect efficiency or compensatory mechanisms. Lastly, environmental factors such as air pollution were not directly assessed, constraining conclusions about the protective role of nasal breathing outdoors. Future studies should investigate ventilatory responses during overground exercise, including diverse populations, and integrate measures such as EMG and perceived exertion ratings. Investigations into the threshold where nasal breathing becomes insufficient at higher intensities, as well as interactions with environmental stressors, are also warranted to develop evidence-based guidelines for breathing strategies in real-world settings.

## 5. Conclusions

This study demonstrated that, in young, recreationally active women, oral and oronasal breathing were associated with lower VT, Ti, Te, and Ttot, along with higher Rf at 10 km/h. At 11 km/h, oral breathing resulted in increased VE and VE/VCO_2_, as well as elevated Rf and reduced Ti, Te, and Ttot during both oral and oronasal breathing. These findings indicate that, within this specific population, exercise intensities exceeding 10 km/h may trigger a compensatory shift toward oral or oronasal breathing to meet increased ventilatory demands. However, this threshold is likely influenced by training status, nasal airflow capacity, and individual physiological characteristics. Therefore, these observations should be interpreted as specific to the studied cohort and not extrapolated as a generalized standard across broader populations.

## Figures and Tables

**Table 1:** Participant Characteristics.

Age (yr)	Height (cm)	Weight (kg)	Physical Activity Level	Athletic Status	Smoking History	Medication Use
24.1 ± 2.1	163.4 ± 3.9	62.7 ± 7.1	Recreationally active (≥2–3 sessions/week)	Non- athlete	None reported	None reported

**Table 2:** Cardiopulmonary responses across breathing conditions and speeds.

Variable	Speed (km)	Nasal	Oral	Oronasal	Inferential Statistics
HR (beats/min)	5	105.6 ± 18.5	104.4 ± 17.8	107.8 ± 10.8	condition (*p* ≤ 0.001, $\eta_{p}^{2}\;$ = 0.971) speed (*p* ≤ 0.001, $\eta_{p}^{2}\;$ = 0.797) interaction (*p* ≤ 0.001, $\eta_{p}^{2}\;$ = 0.463)
	7	131.4 ± 13.1	129.4 ± 19.4	128.0 ± 13.6	
	9	155.2 ± 11.7	151.9 ± 16.2	154.6 ± 8.4	
	10	170.2 ± 8.7	166.0 ± 12.6	170.6 ± 6.9	
	11	179.3 ± 6.3	174.2 ± 8.1	177.9 ± 8.0	
VO_2_ (mL/kg/min)	5	17.6 ± 2.8	16.0 ± 1.7	17.6 ± 3.6	condition (*p* ≤ 0.001, $\eta_{p}^{2}\;$ = 0.970) speed (*p* ≤ 0.001, $\eta_{p}^{2}\;$ = 0.856) interaction (*p* ≤ 0.001, $\eta_{p}^{2}\;$ = 0.616)
	7	28.2 ± 4.0	25.8 ± 3.3	27.8 ± 4.4	
	9	35.5 ± 5.6	33.8 ± 3.9	35.5 ± 5.7	
	10	39.4 ± 5.1	36.5 ± 5.0	38.5 ± 4.8	
	11	41.7 ± 4.8	39.1 ± 6.9	39.9 ± 5.9	
VE/VCO_2_	5	31.1 ± 2.7	31.6 ± 1.8	30.4 ± 3.6	condition (*p* = 0.004, $\eta_{p}^{2}\;$ = 0.549) speed (*p* = 0.115, $\eta_{p}^{2}\;$ = 0.226) interaction (*p* = 0.305, $\eta_{p}^{2}\;$ = 0.148)
	7	29.7 ± 3.3	30.6 ± 1.8	28.8 ± 3.7	
	9	28.6 ± 3.3	29.8 ± 1.8	28.2 ± 4.0	
	10	27.6 ± 2.9	29.9 ± 1.2	28.4 ± 3.3	
	11	27.2 ± 2.9 *	30.3 ± 1.4 *	29.1 ± 2.8	

Values are Mean ± Standard deviation. HR—heart rate; VO_2_—oxygen uptake; VE/VCO_2_—minute ventilation/carbon dioxide production. * Significantly different from nasal breathing.

**Table 3:** Ventilatory responses across breathing conditions and speeds.

Variable	Speed (km)	Nasal	Oral	Oronasal	Inferential Statistics
VT (L)	5	0.9 ± 0.2	0.9 ± 0.2	0.9 ± 0.2	condition (*p* ≤ 0.001, $\eta_{p}^{2}\;$ = 0.909) speed (*p* ≤ 0.001, $\eta_{p}^{2}\;$ = 0.721) interaction (*p* ≤ 0.001, $\eta_{p}^{2}\;$ = 0.466)
	7	1.3 ± 0.3	1.3 ± 0.3	1.2 ± 0.3	
	9	1.5 ± 0.4	1.4 ± 0.2	1.5 ± 0.3	
	10	1.6 ± 0.3	1.5 ± 0.3 *	1.5 ± 0.2 *	
	11	1.6 ± 0.3	1.5 ± 0.3	1.6 ± 0.3	
Rf (breaths/min)	5	28.4 ± 5.7	27.1 ± 4.0	29.0 ± 5.6	condition (*p* ≤ 0.001, $\eta_{p}^{2}\;$ = 0.897) speed (*p* ≤ 0.001, $\eta_{p}^{2}\;$ = 0.757) interaction (*p* = 0.551, $\eta_{p}^{2}\;$ = 0.110)
	7	33.4 ± 8.4	32.2 ± 5.6	33.6 ± 8.6	
	9	36.8 ± 9.6	39.4 ± 5.3	39.9 ± 10.9	
	10	40.4 ± 8.6	44.3 ± 7.3 *	45.2 ± 9.5 *	
	11	44.1 ± 8.6	49.8 ± 7.9 *	49.6 ± 11.8 *	
VE (L/min)	5	25.0 ± 2.2	23.2 ± 3.4	25.6 ± 5.4	condition (*p* ≤ 0.001, $\eta_{p}^{2}\;$ = 0.980) speed (*p* ≤ 0.001, $\eta_{p}^{2}\;$ = 0.915) interaction (*p* ≤ 0.001, $\eta_{p}^{2}\;$ = 0.427)
	7	39.0 ± 3.8	38.8 ± 4.3	39.6 ± 7.4	
	9	52.5 ± 3.6	54.8 ± 7.8	55.2 ± 9.0	
	10	61.6 ± 4.8	64.0 ± 7.8	65.5 ± 9.9	
	11	67.2 ± 7.9	73.7 ± 7.0 *	74.8 ± 10.6 *	

Values are Mean ± Standard deviation. VT—tidal volume; Rf—respiratory frequency; VE—minute ventilation. * Significantly different from nasal breathing.

**Table 4:** Breathing timing responses across breathing conditions and speeds.

Variable	Speed (km)	Nasal	Oral	Oronasal	Inferential Statistics
Ti (s)	5	1.0 ± 0.3	1.0 ± 0.1	0.9 ± 0.2	condition (*p* ≤ 0.001, $\eta_{p}^{2}\;$ = 0.794) speed (*p* ≤ 0.001, $\eta_{p}^{2}\;$ = 0.510) interaction (*p* = 0.740, $\eta_{p}^{2}\;$ = 0.084)
	7	0.9 ± 0.2	0.9 ± 0.1	0.9 ± 0.2	
	9	0.9 ± 0.2	0.8 ± 0.1	0.8 ± 0.3	
	10	0.8 ± 0.2	0.7 ± 0.1	0.7 ± 0.2 *	
	11	0.7 ± 0.2	0.6 ± 0.1 *	0.6 ± 0.2 *	
Te (s)	5	1.3 ± 0.3	1.5 ± 0.3	1.3 ± 0.3	condition (*p* ≤ 0.001, $\eta_{p}^{2}\;$ = 0.923) speed (*p* ≤ 0.001, = 0.779) interaction (*p* = 0.001, $\eta_{p}^{2}\;$= 0.383)
	7	1.0 ± 0.3	1.1 ± 0.2	1.0 ± 0.3	
	9	0.9 ± 0.2	0.8 ± 0.1	0.9 ± 0.3	
	10	0.8 ± 0.1	0.7 ± 0.1	0.7 ± 0.2 *	
	11	0.7 ± 0.1	0.6 ± 0.1 *	0.7 ± 0.2 *	
Ttot (s)	5	2.3 ± 0.5	2.4 ± 0.4	2.2 ± 0.5	condition (*p* ≤ 0.001, $\eta_{p}^{2}\;$ = 0.895) peed (*p* ≤ 0.001, $\eta_{p}^{2}\;$ = 0.739) interaction (*p* = 0.070, $\eta_{p}^{2}\;$ = 0.218)
	7	2.0 ± 0.5	2.0 ± 0.3	2.0 ± 0.5	
	9	1.8 ± 0.5	1.6 ± 0.2	1.7 ± 0.5	
	10	1.6 ± 0.3	1.4 ± 0.2	1.4 ± 0.4 *	
	11	1.4 ± 0.3	1.3 ± 0.2 *	1.3 ± 0.3 *	

Values are Mean ± Standard deviation. Ti—inspiratory time; Te—expiratory time; Ttot—total respiratory cycle time. * Significantly different from nasal breathing.

## Data Availability

The data presented in this study are available on request from the corresponding author. The data are not publicly available due to the protection of personal information.

## Abbreviations

The following abbreviations are used in this manuscript:

CO_2_	Carbon dioxide
HR	Heart rate
Rf	Respiratory frequency
Te	Expiratory time
Ti	Inspiratory time
Ttot	Total respiratory cycle time
VE	Minute ventilation
VE/VCO_2_	Minute ventilation/carbon dioxide production
VO_2_	Oxygen uptake
VT	Tidal volume
